# All Hands on Deck! A Challenging Case with a Multidisciplinary Team Using Negative-pressure Wound Therapy as the Cornerstone for Treatment

**DOI:** 10.7759/cureus.3621

**Published:** 2018-11-21

**Authors:** Michael N Desvigne, Brian Lipton, Debra McClary, Krista Montgomery, Kurt Holifield, Kari Day

**Affiliations:** 1 Plastic Surgery, Abrazo Arrowhead Hospital, Glendale, USA; 2 Surgery, Abrazo Arrowhead Hospital, Glendale, USA

**Keywords:** npwt, npwt with instillation, npwt with instillation, npwt-id, cleanse, cleanse choice, grossly contaminated wound, abdomen, surgical alternative, nonsurgical candidate

## Abstract

The use of negative-pressure wound therapy (NPWT) has become the new standard of care for complex wounds. NPWT with instillation (NPWTi) has been shown to assist wound progression in a variety of wound types in an acute hospital setting with increased progression toward healing.

We present the case of a 70-year-old male with Crohn's disease, who had post-operative life-threatening complications following hernia repair. His complex abdominal wound and a high-output fistula required the assistance of an entire clinical team.

The multidisciplinary team's approach toward the patient was equivalent to the team's approach to the complex wound: "All Hands On Deck!" The cornerstone of our management was NPWT, specifically NPWTi. Instillation therapy was initiated. Complex foam application and innovative strategies to keep a grossly contaminated wound from becoming the final straw to a patient's demise appeared our greatest challenge.

NPWTi was utilized and optimized, where every type of foam, bridge, and securement was needed to gain success. This patient's progress could be wholly attributed to the commitment and experience of a group of care providers who were led by their knowledge and experience in NPWT in the most challenging circumstances.

## Introduction

Negative-pressure wound therapy (NPWT; V.A.C. Therapy, KCI, An Acelity Company, San Antonio, TX, USA) is a disruptive technology that has changed the standard of care for the treatment of complex wounds. More recently, the addition of instillation and dwell (NPWTi-d) has combined the benefits of NPWT with controlled delivery of topical solutions and suspensions over the wound bed, thereby reducing edema and promoting perfusion and granulation tissue formation. When used with novel dressings such as reticulated open-celled foam with through holes (ROCF-CC; V.A.C. VERAFLO CLEANSE CHOICE Dressing, KCI, An Acelity Company), the combination facilitates the removal of thick exudates and infectious materials, such as fibrin and slough, providing a wound-cleansing option for clinicians when surgical debridement must be delayed or is not possible or appropriate [1-3].

We present a case study of a 70-year-old male with an enterocutaneous fistula resulting from post-operative complications following incarcerated hernia repair.

This challenging case required a multidisciplinary team of wound clinicians, general surgeons, colorectal surgeons, and plastic surgeons to improvise and create solutions. Using NPWT as the cornerstone of treatment, the patient was successfully managed with NPWTi throughout the continuum of care, utilizing traditional NPWT, and finally negative-pressure therapy (NPT) for incisional management at the time of closure.

In this study, our goal is to discuss a case that was treated successfully only because of the team approach, creative thinking, and the use of NPWT, both traditional and advanced therapies, to manage a critically ill patient.

## Case presentation

**Figure 1:**
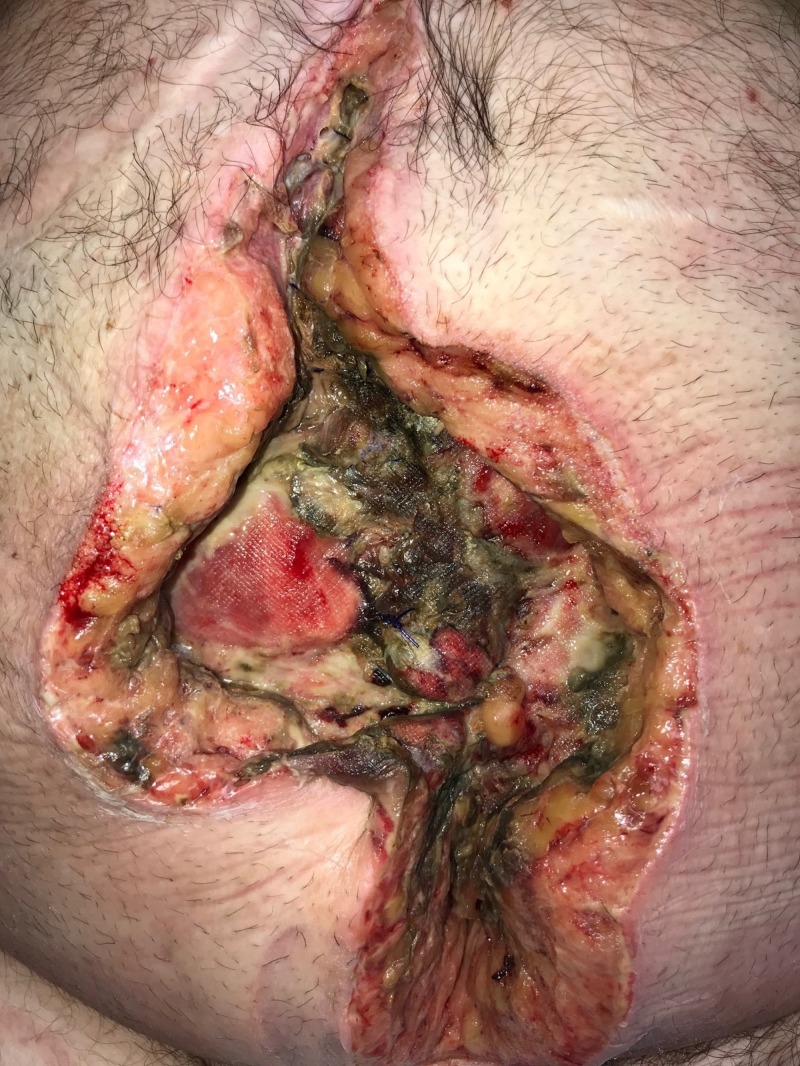
Day 1: Grossly contaminated wound Wound with significant necrotic debris; no gross evidence of fistula or bowel exposure; patient considered to have "too high" risk for surgical debridement

**Figure 2:**
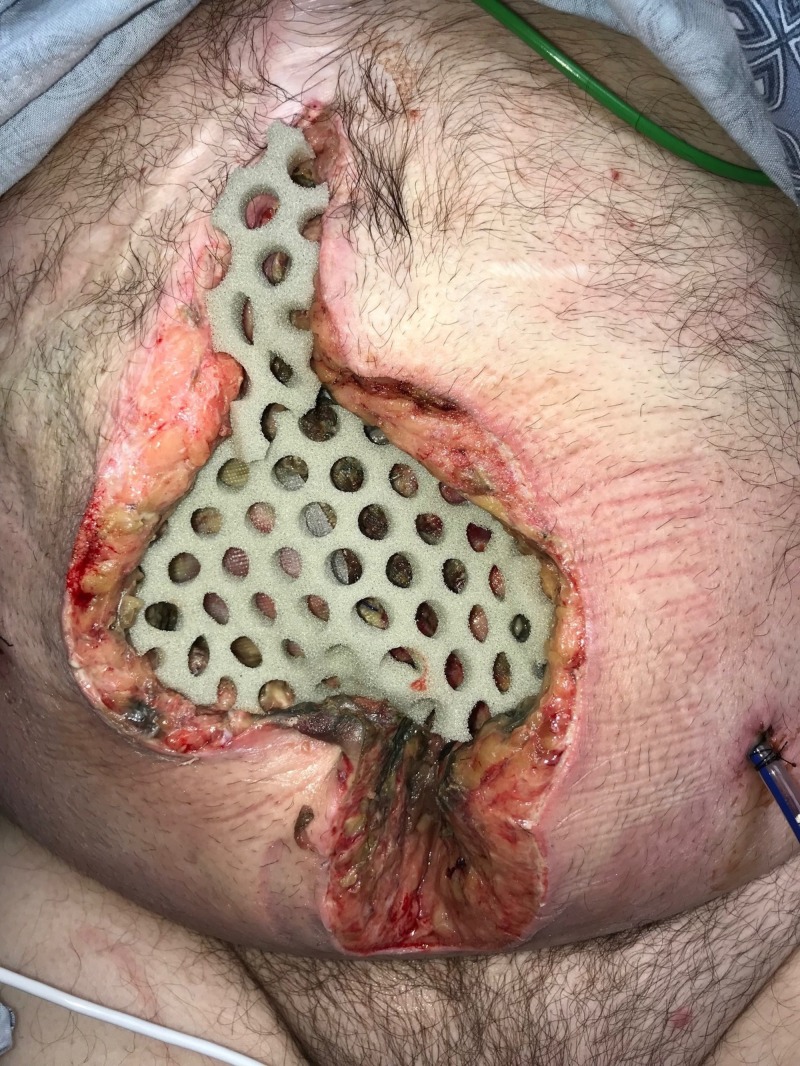
Day 1: NPWTi-d with ROCF-CC placement NPWTi-d: negative-pressure wound therapy with instillation and dwell; ROCF-CC: reticulated open-celled foam with through holes

**Figure 3:**
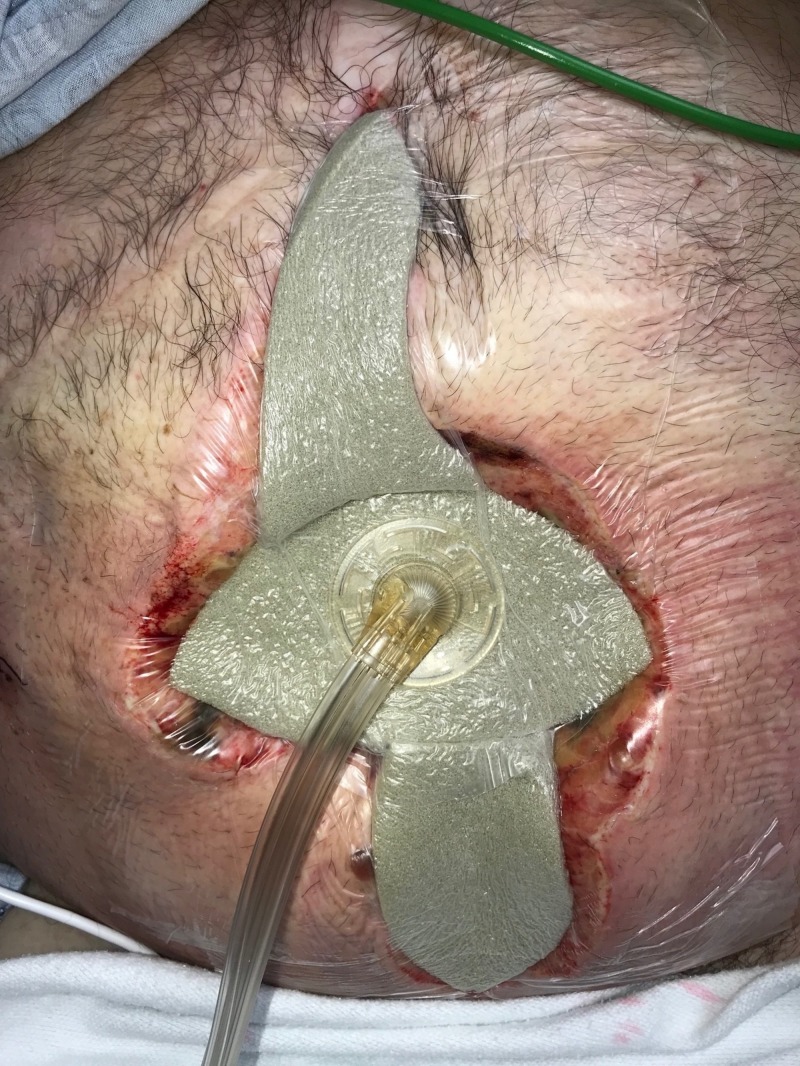
Day 1: NPWTi-d with ROCF-CC placement NPWTi-d: negative-pressure wound therapy with instillation and dwell; ROCF-CC: reticulated open-celled foam with through holes

A 70-year-old gentleman with Crohn's disease presented with an incarcerated ventral hernia that required urgent surgical intervention. Following repair, he returned two weeks later with an acutely distended abdomen and early sepsis. Emergent laparotomy revealed a contaminated abdomen with a small bowel perforation in a segment of the bowel in Crohn's disease. He returned to the operating room for exploration and bowel resection and primary anastomosis. The incision was left open due to severe contamination. In the operating room, instillation therapy was initiated with NPWTi-d along with ROCF-CC with normal saline with a 20-minute dwell time and continuous negative pressure at -125 mmHg. ROCF-CC was chosen for the mechanism of action to "fracture" the necrotic debris and perhaps avoid the need for an additional operative intervention. Normal saline was chosen to reduce bacterial burden and contain the cost of care (Figures 1-3).

**Figure 4:**
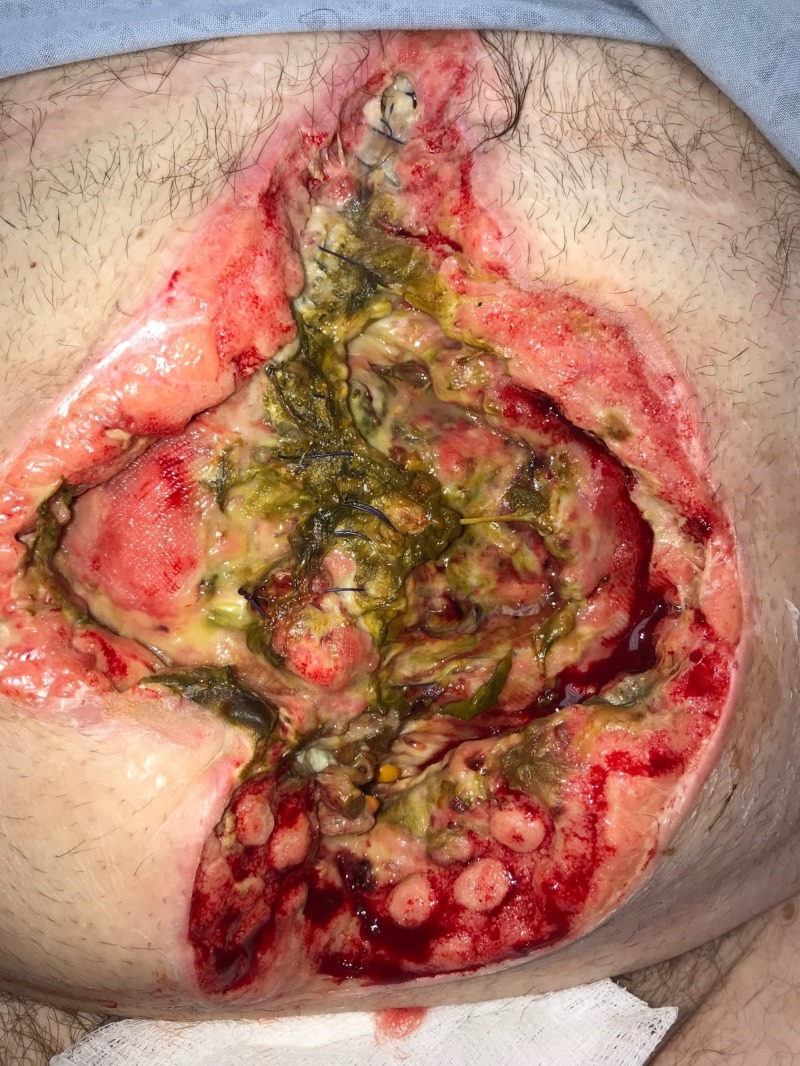
Day 4: Wound improved after three days of NPWTi-d with ROCF-CC Wound with less necrotic debris and early granulation tissue NPWTi-d: negative-pressure wound therapy with instillation and dwell; ROCF-CC: reticulated open-celled foam with through holes

Following three days of NPWTi-d with ROFC-CC, there was significant wound improvement with increasing granulation tissue and less necrotic debris (Figure 4).

However, the patient developed a leak at the anastomosis requiring another emergent operation. During this operation, which was performed as "damage control", the general surgeon and colorectal surgeon agreed it would be best to avoid another anastomosis and the small bowel was delivered to the skin as a controlled fistula. 

The wound team and plastic surgery were consulted to assist in managing a contaminated abdominal midline incision and wound with a high-output fistula in a patient who was now critically ill with early sepsis.

**Figure 5:**
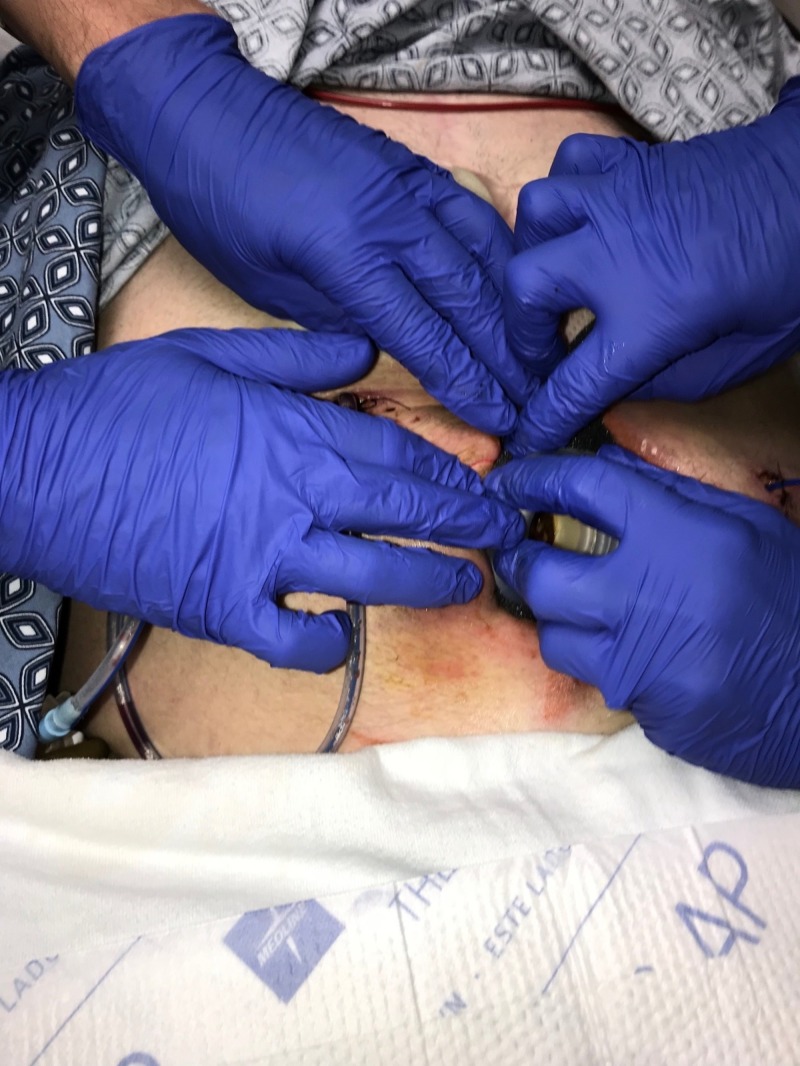
All hands on deck Complex dressing change requiring team approach

The team's approach to the patient was as integrated as the team's approach to the complex wound. "All Hands On Deck!" (Figure 5).

**Figure 6:**
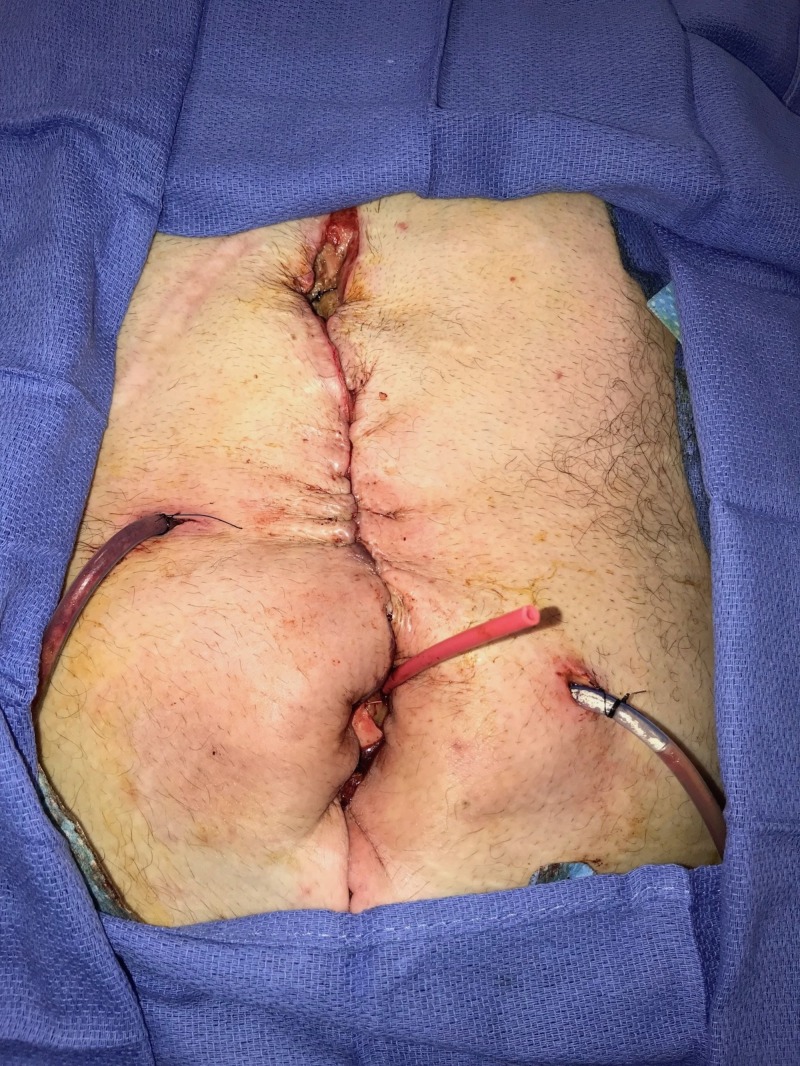
Day 7: Abdomen partly closed Small bowel leak diagnosed; patient returned to operating room; incision partly closed following fistula creation.

**Figure 7:**
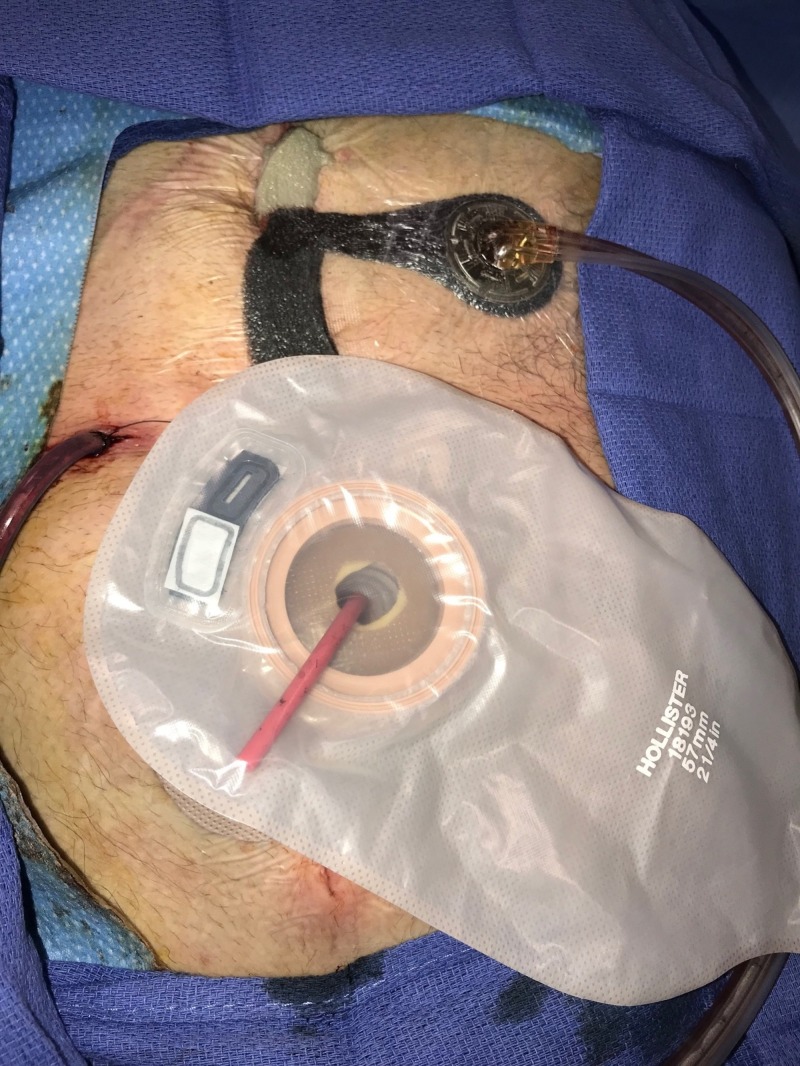
Day 7: NPWTi-d with ROCF-CC and fistula management Combination foam utilized for NPWTi-d NPWTi-d: negative-pressure wound therapy with instillation and dwell; ROCF-CC: reticulated open-celled foam with through holes

The cornerstone of our management was NPWT. Instillation therapy was initiated with NPWTi-d along with ROCF-CC in the operating room with normal saline with a 20-minute dwell time and continuous negative pressure at -125 mmHg (Figures 6-7).

**Figure 8:**
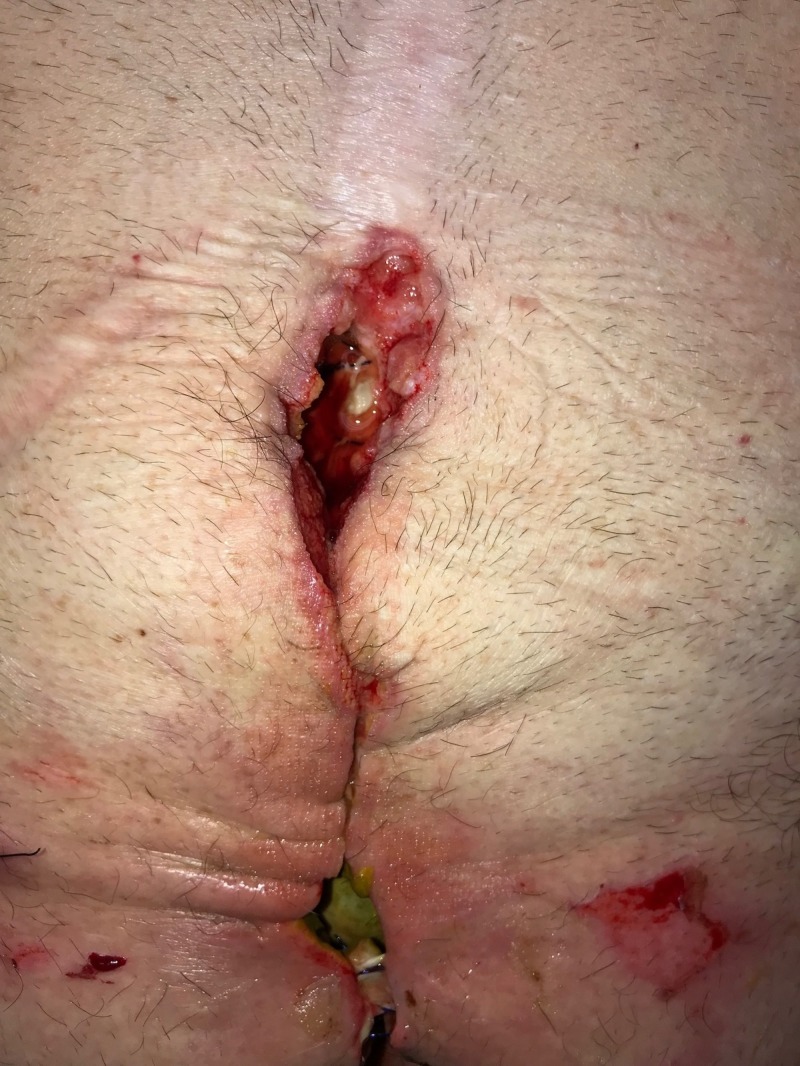
Day 11: Wound improving with NPWTi-d with ROCF-CC NPWTi-d: negative-pressure wound therapy with instillation and dwell; ROCF-CC: reticulated open-celled foam with through holes

Complex foam application, bridging, and innovative strategies to keep a grossly contaminated wound from becoming the final straw to a patient's demise appeared our greatest challenge. Ostomy paste, rings, colloid, and barriers were utilized. Other items such as infant bottle nipples and condom catheters were also required. The team worked side by side to create dressing strategies to deliver NPWTi in the face of a high-output fistula to control contamination and allow primary healing. Despite the high output, the wounds improved (Figure 8).

NPWTi was the cornerstone of our management strategy, wherein every type of foam, bridge, and securement was needed to gain success. As the patient's clinical status improved, he was transitioned from total parenteral nutrition (TPN) to an oral diet. However, the high-output fistula resulted in an increasing wound size that required continued NPWT which was transitioned from NPWTi-d to NPWTi-d to NPWT. (Figures 9-19).

**Figure 9:**
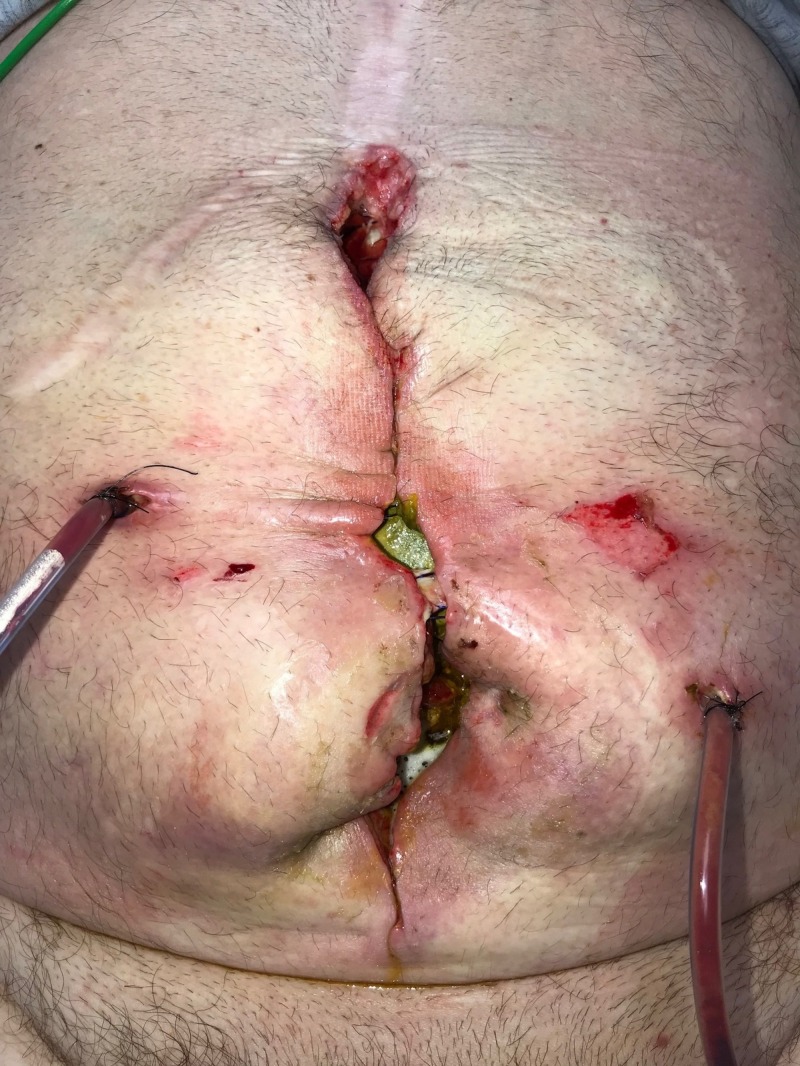
Day 11: Distal wound expanding secondary to fistula leakage Incisional dehiscence secondary to periwound irritation from fistula leakage despite protective barriers applied to skin; proximal wounds with less necrotic debris NPWTi-d: negative-pressure wound therapy with instillation and dwell; ROCF-CC: reticulated open-celled foam with through holes

**Figure 10:**
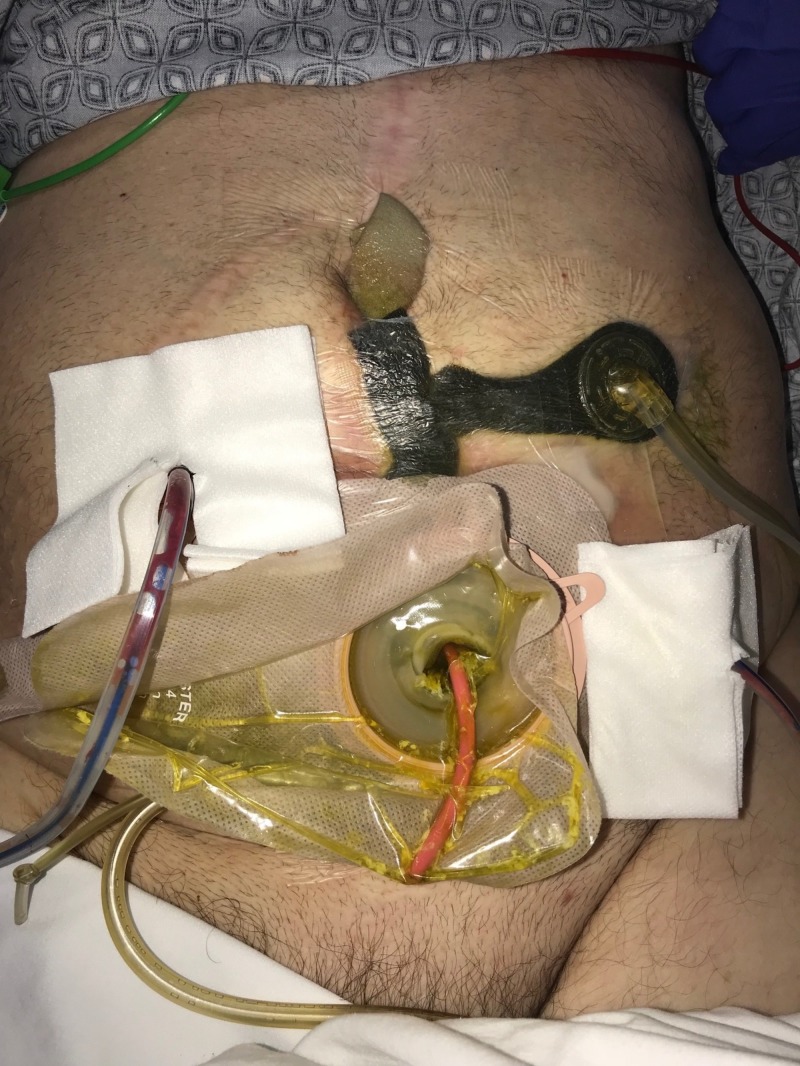
Day 11: Ostomy applied and NPWTi-d continued Fistula controlled with ostomy appliance; NPWTi-d with combination foam therapy continued NPWTi-d: negative-pressure wound therapy with instillation and dwell; ROCF-CC: reticulated open-celled foam with through holes

**Figure 11:**
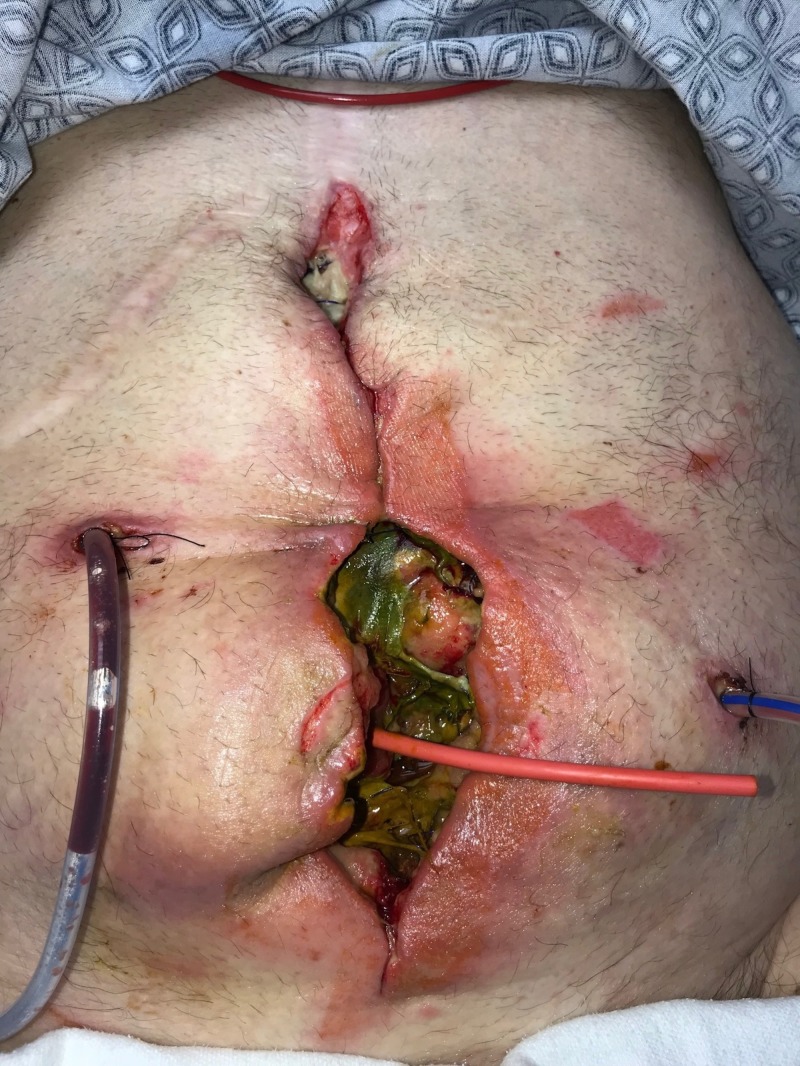
Day 14: Fistula leakage causing further dehiscence

**Figure 12:**
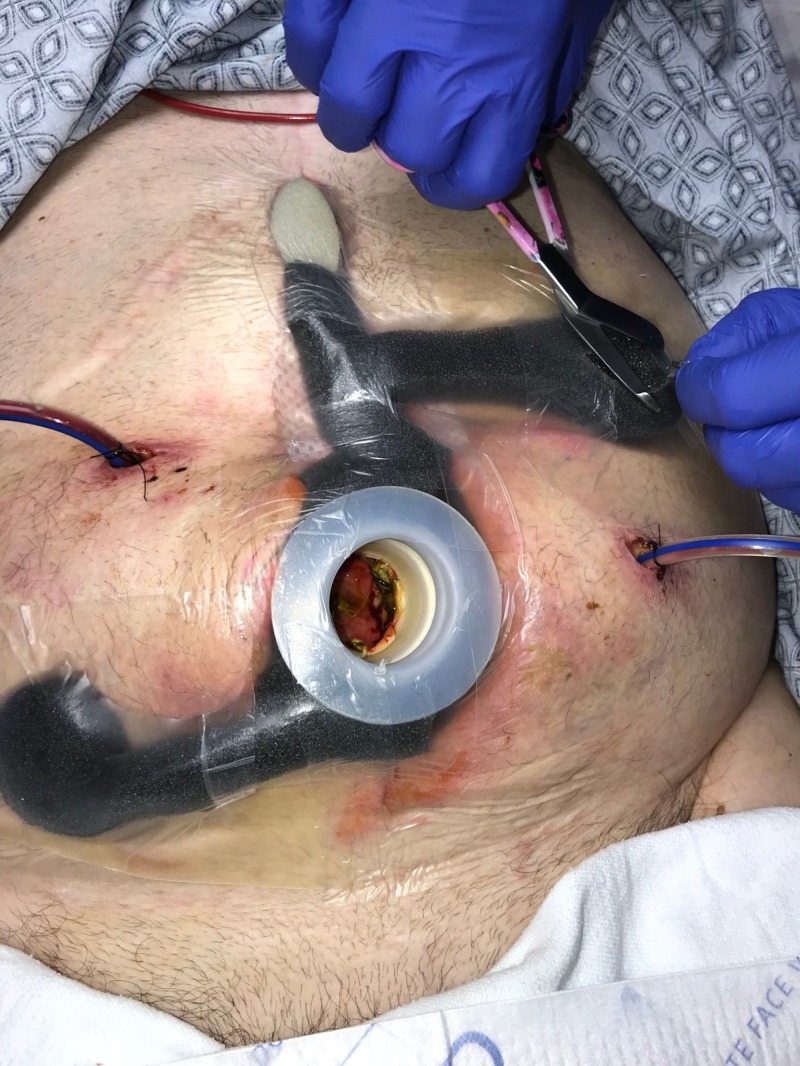
Day 14: Fistula management with NPWTi-d NPWTi-d: negative-pressure wound therapy with instillation and dwell

**Figure 13:**
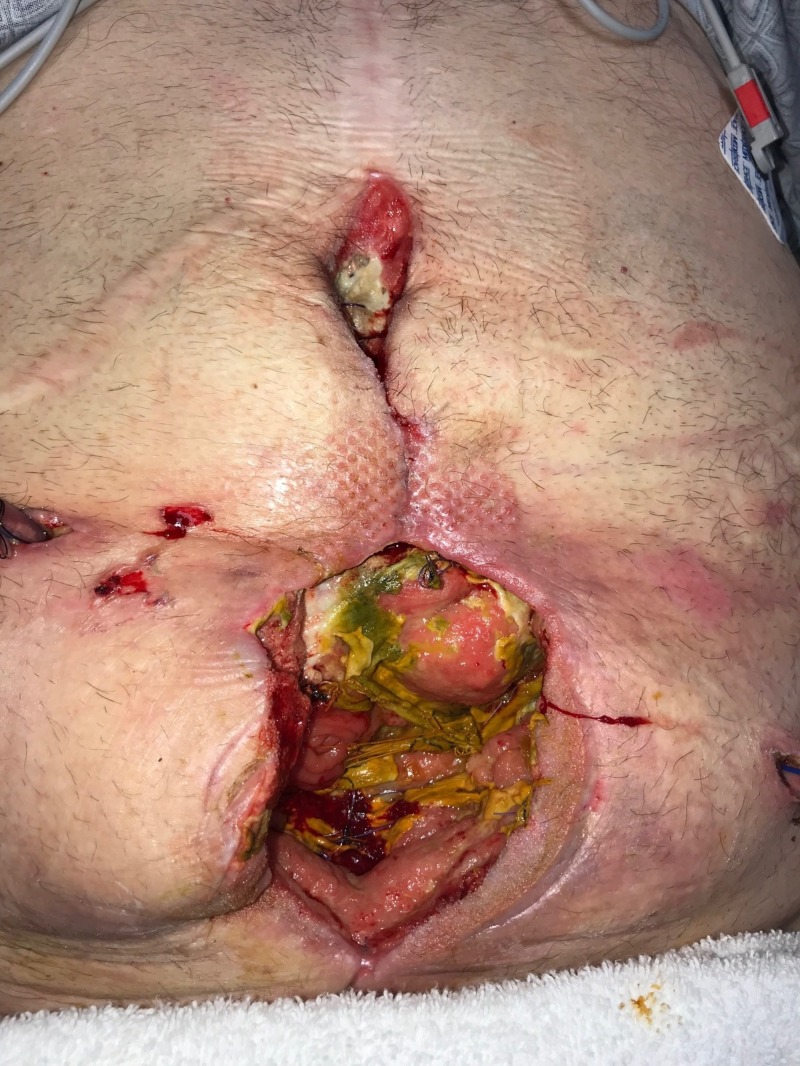
Day 18: Wound larger secondary to high-output fistula

**Figure 14:**
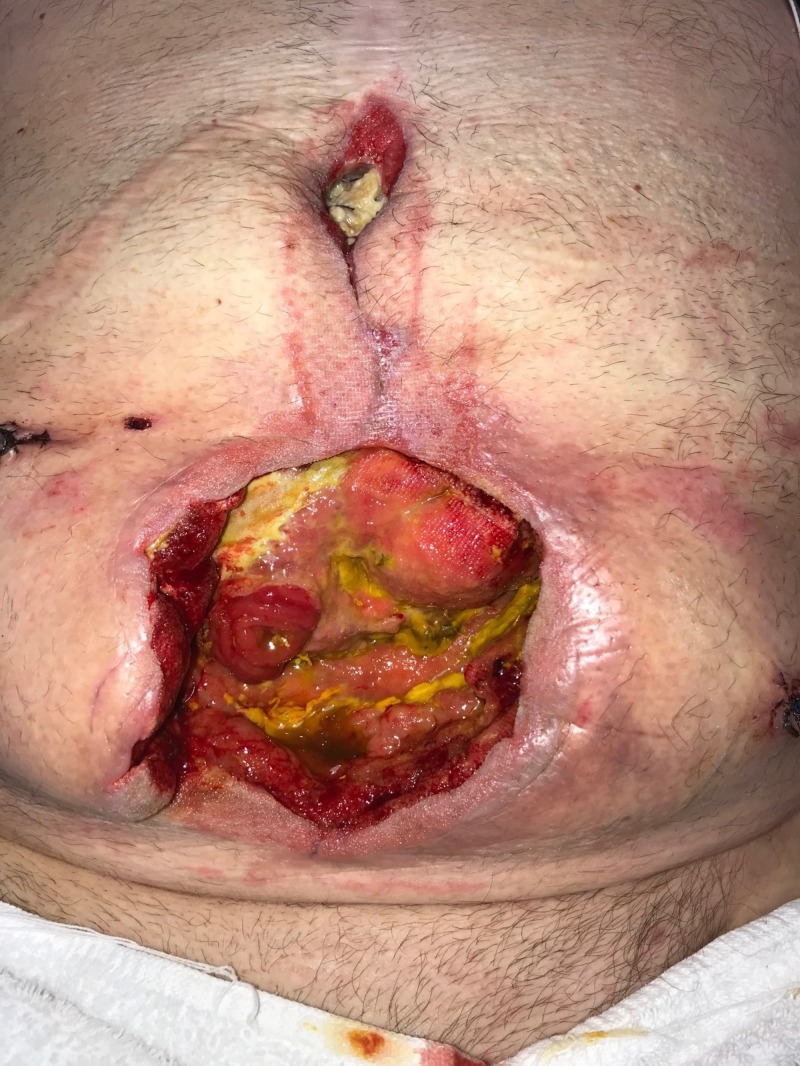
Day 21

**Figure 15:**
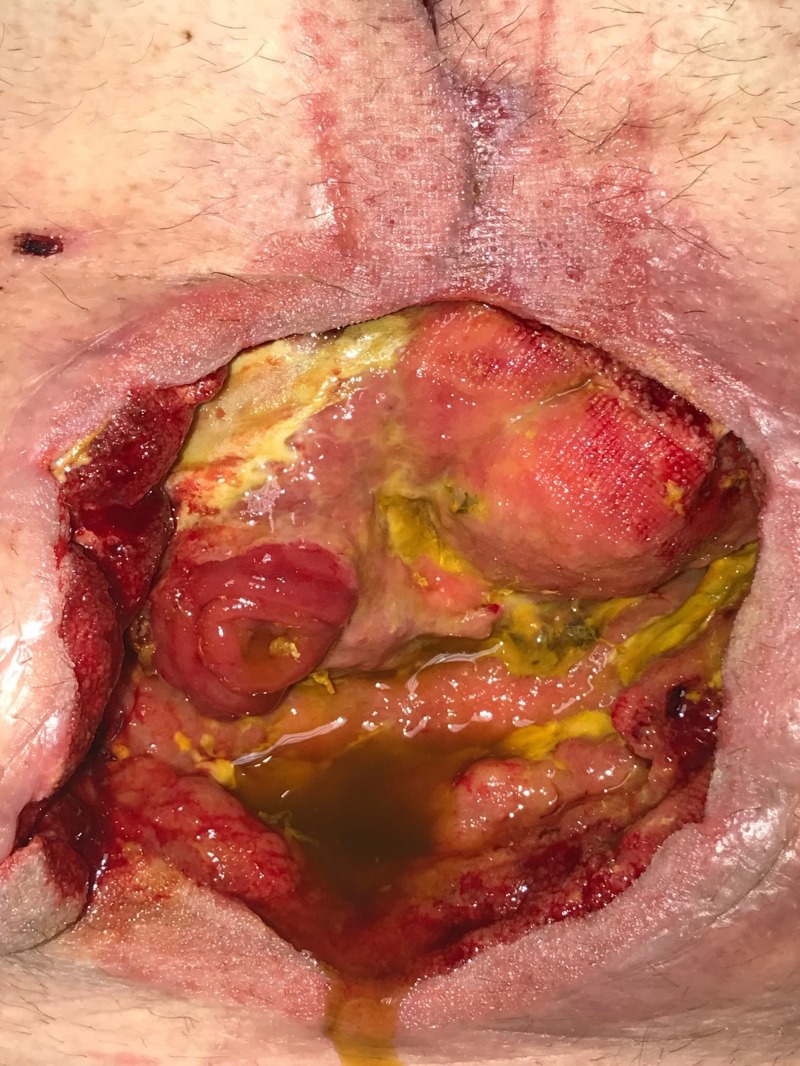
Day 21: High-output fistula

**Figure 16:**
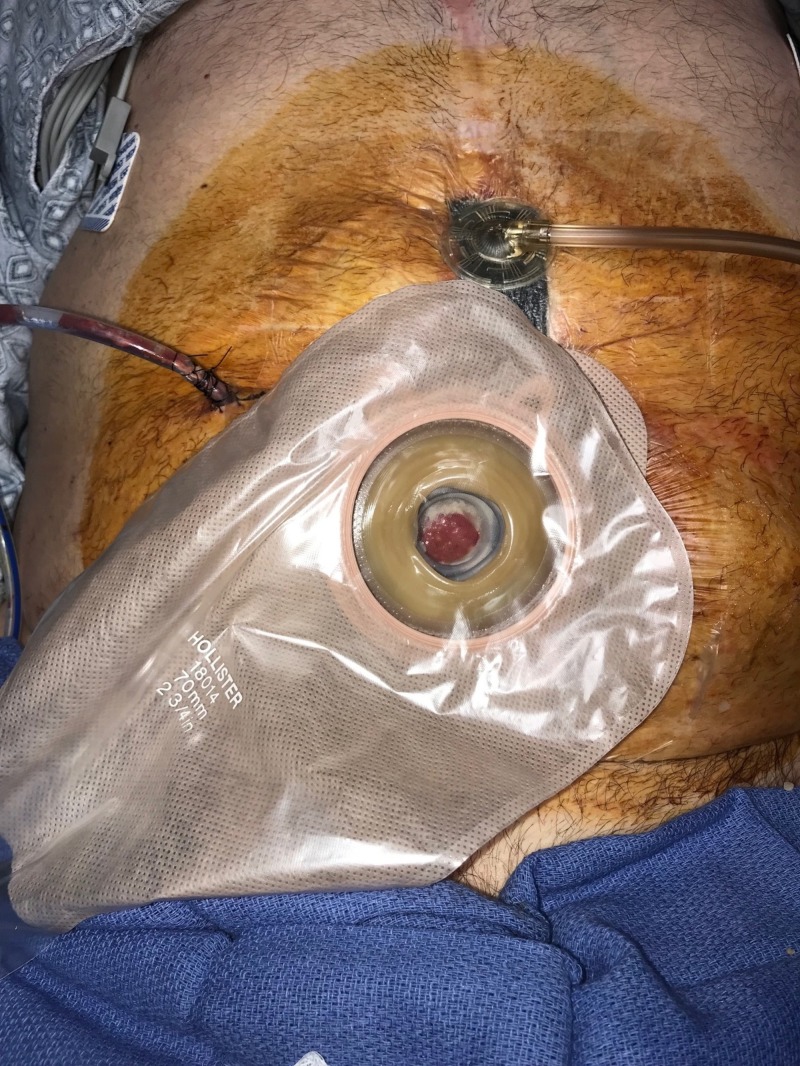
Day 21: NPWTi-d continued NPWTi-d: negative-pressure wound therapy with instillation and dwell

**Figure 17:**
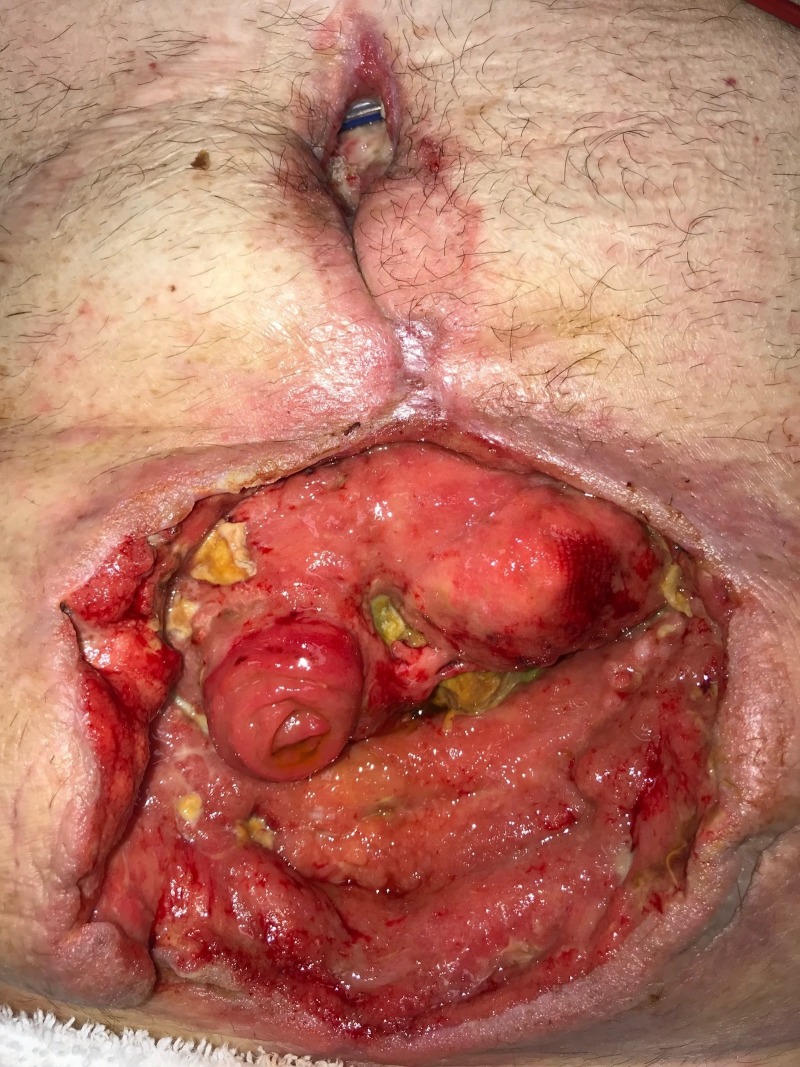
Four weeks: wound bed improvement Fistula stomatized; wound bed with healthy granulation tissue present

**Figure 18:**
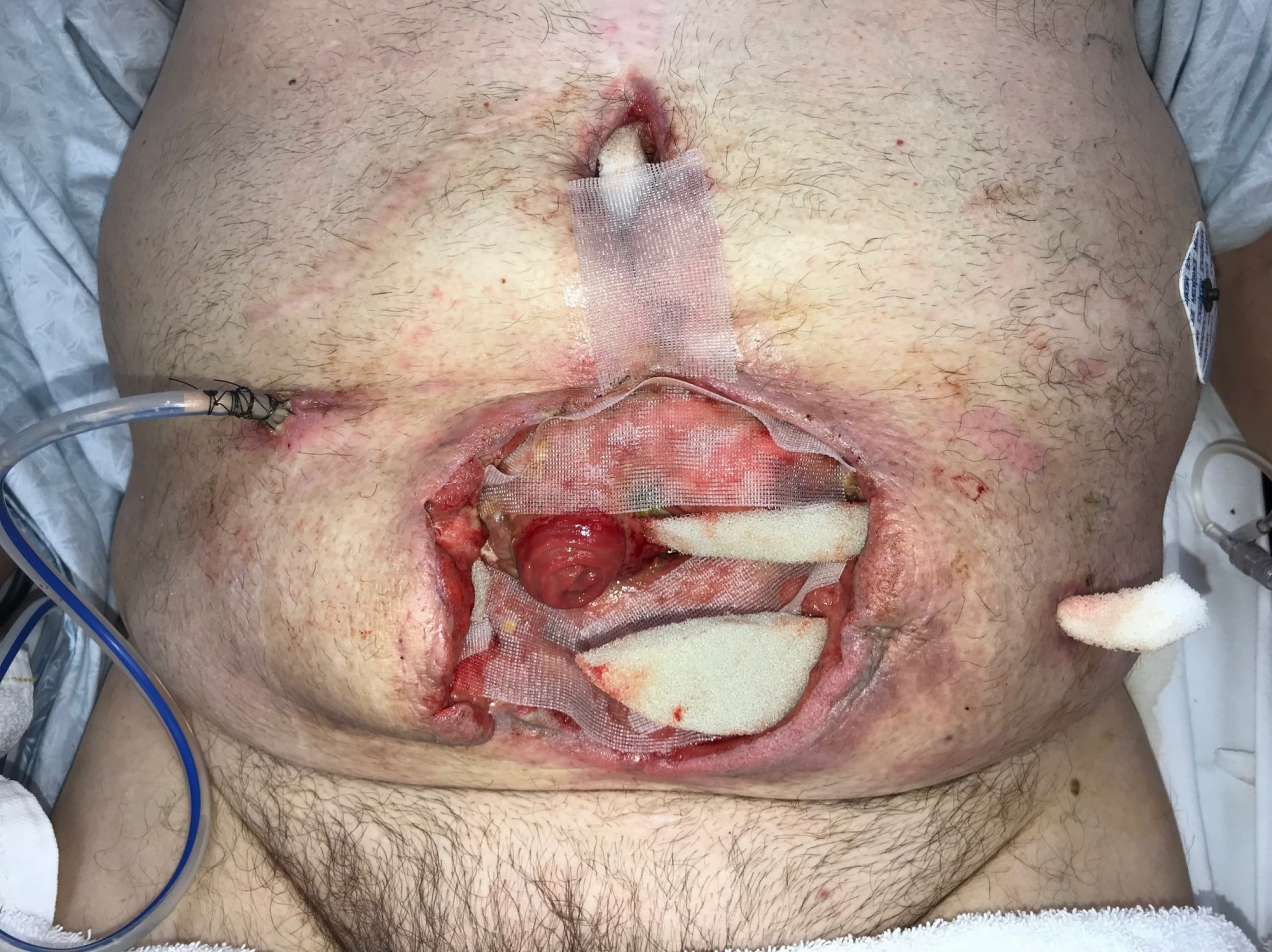
Four weeks: Complex NPWT application White foam placed in undermined areas; combination of NPWT and NPWTi-d NPWTi-d: negative-pressure wound therapy with instillation and dwell

**Figure 19:**
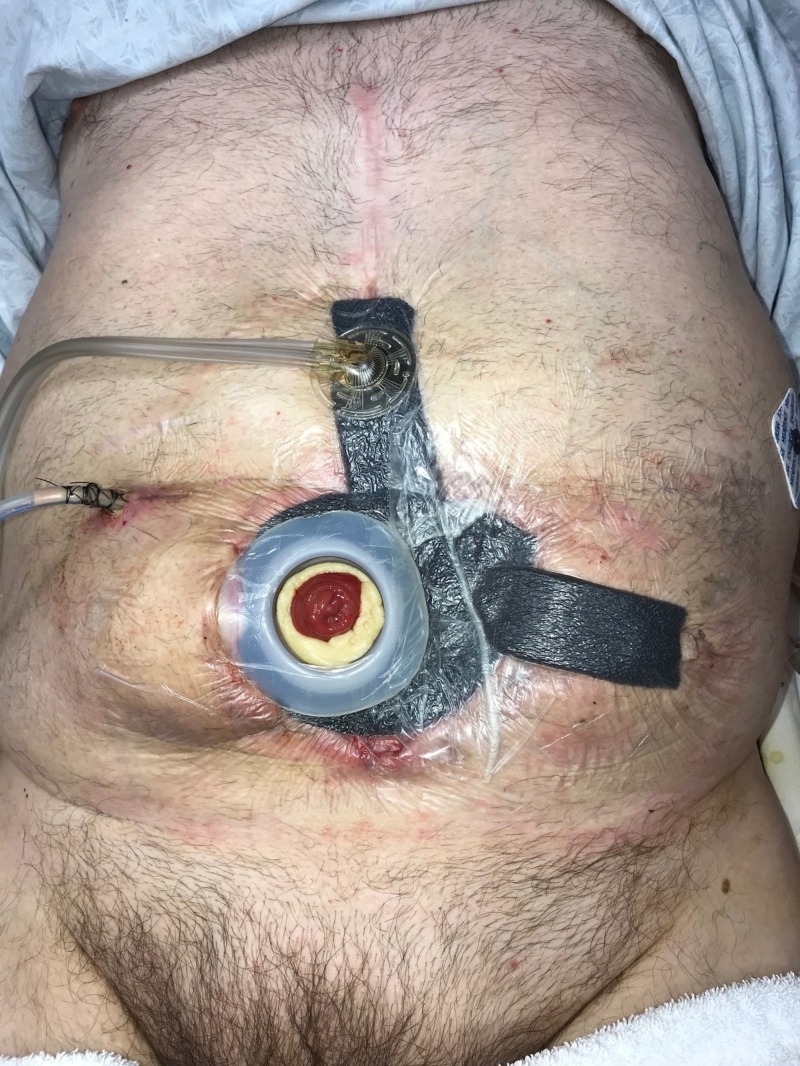
Four weeks: NPWT continued Transitioned from NPWTi-d to NPWT NPWTi-d: negative-pressure wound therapy with instillation and dwell

**Figure 20:**
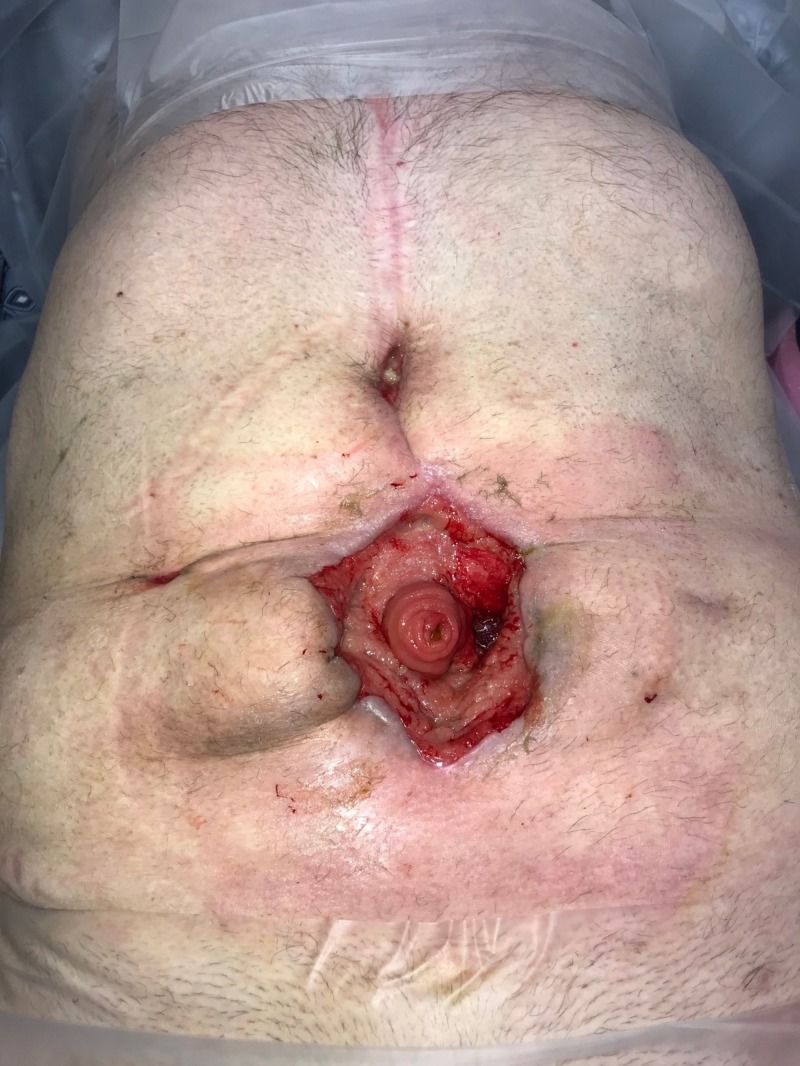
Eight weeks: Healthy wound bed Wound optimal for closure

**Figure 21:**
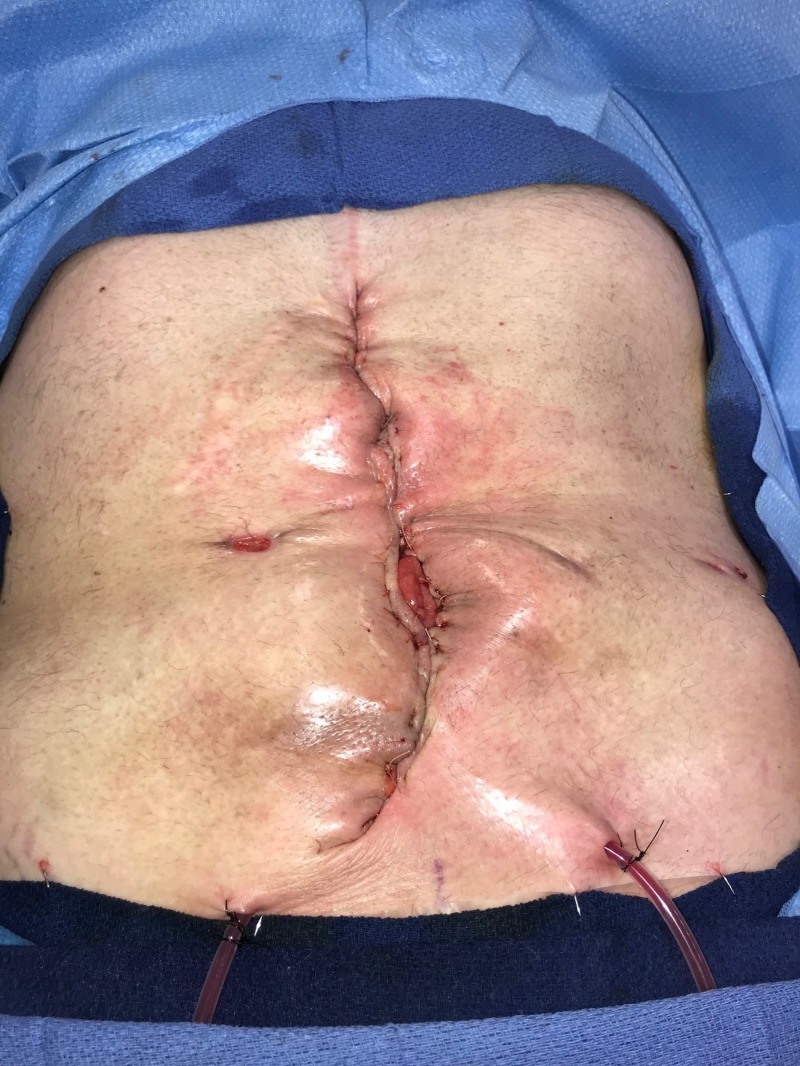
Eight weeks: Abdomen closed Fistula brought to the skin surface, but with minimal mobility

**Figure 22:**
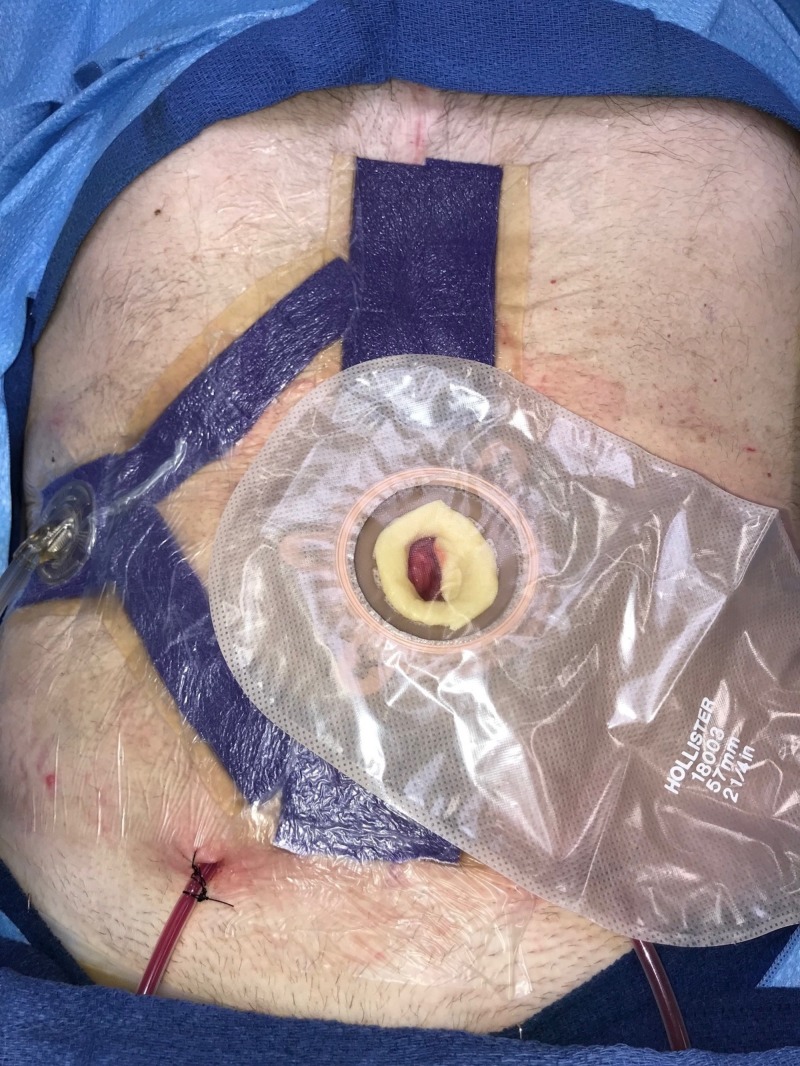
Eight weeks: Prevena Plus dressing (KCI, An Acelity Company, San Antonio, TX, USA) placed for incisional management -125 mmHg continuous negative pressure therapy for seven days

Once his clinical status improved, he was returned to the operating room for closure (Figures 20-22).

Despite a successful closure, the challenge of fistula contamination continued. The initial closure was complicated by the inversion and disruption of the mucocutaneous sutures causing severe contamination to the distal incision. Revision required local tissue rearrangement and fasciocutaneous flaps to create a flatter, more appropriate topography of the stoma such that the fistula contents could be above the skin level.

Following this procedure, it was thought that incisional management could not be instituted due to fear of leakage under the dressing with secondary contamination. Unfortunately, two days after closure, the local flap dehisced and revision was required. During the operation, the local tissue rearrangement was redone, and the distal incision was managed with NPT as incisional management at a continuous negative pressure of -125 mmHg.

**Figure 23:**
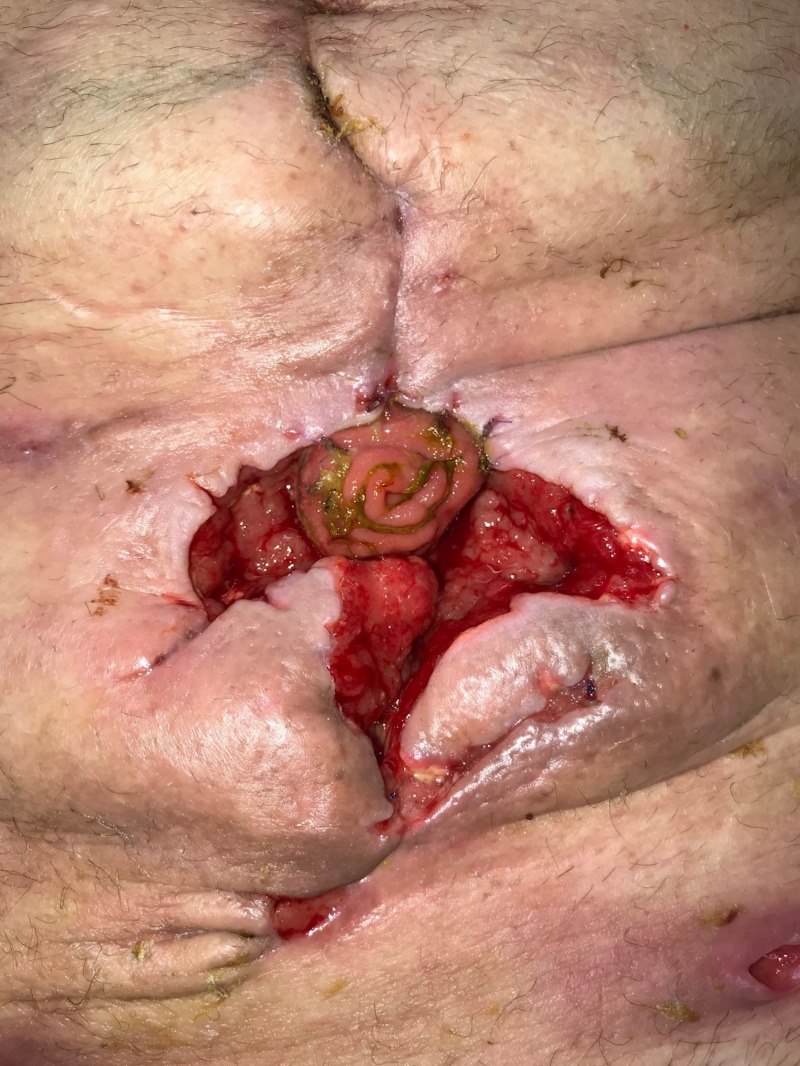
12 weeks following NPWT: Healthy wound bed achieved Wound optimal for transitional level of care NPWT: negative-pressure wound therapy

The high-output fistula continued with leakage and contamination, resulting in recurrent break down of the distal incision. NPWT was reinstituted to assist with fistula management. Due to the continued high output, it was elected to hold on any further attempt at surgical closure and depend solely on wound and fistula management. Fortunately, the patient's clinical progress continued to improve. The wound bed continued to improve with a healthy granulating tissue filling in the defect (Figure 23).

**Figure 24:**
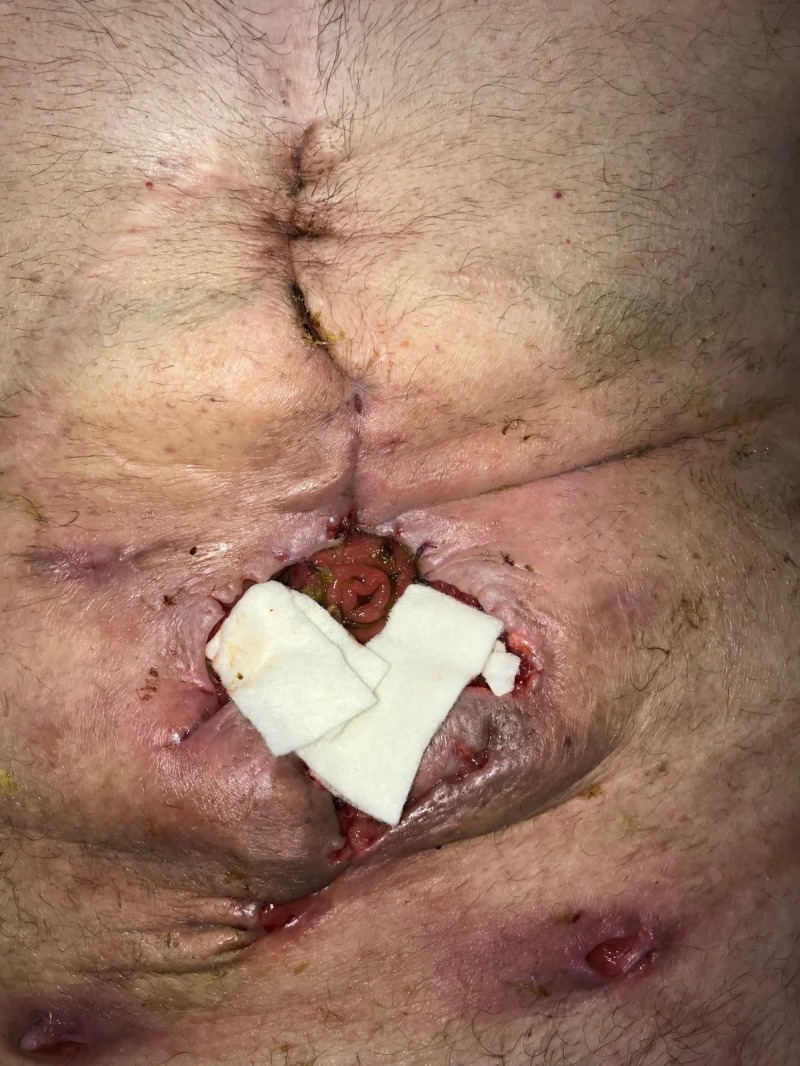
12 weeks: Advanced dressings placed with ORC/collagen/silver dressings, Promogran Prisma (KCI, An Acelity Company, San Antonio, TX, USA) Dressings stacked to achieve a flat surface optimal for ostomy appliance ORC: oxidized regenerated cellulose

**Figure 25:**
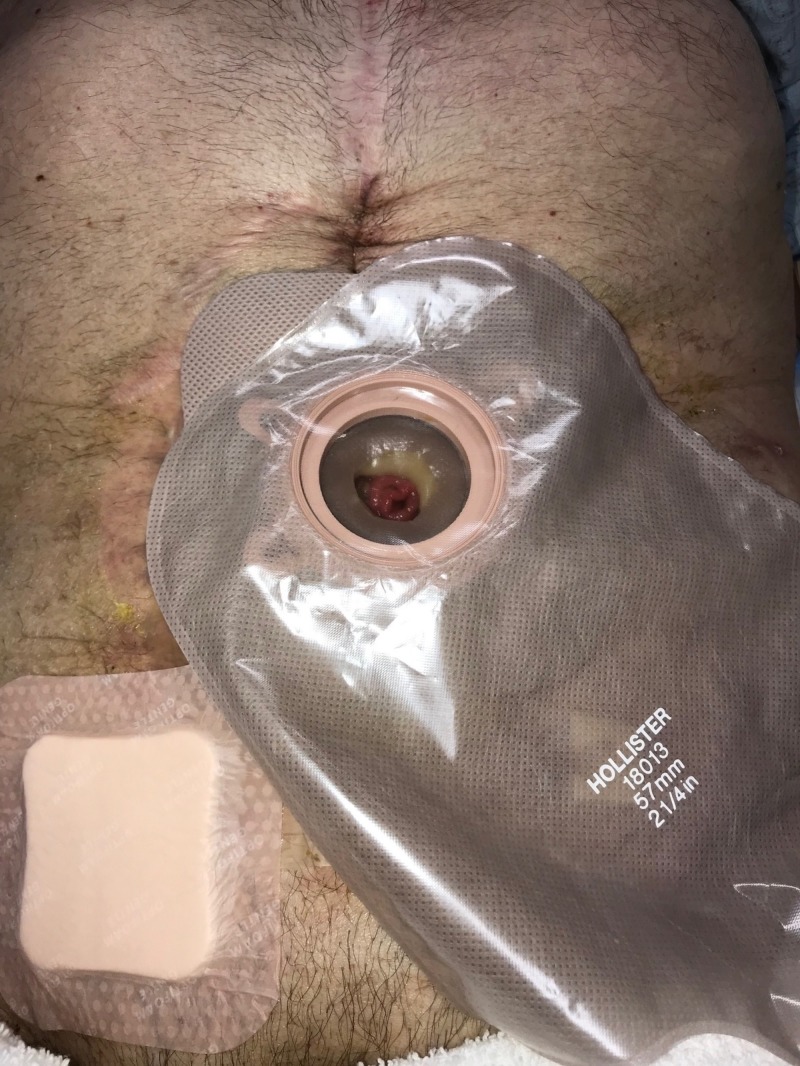
12 weeks: Advanced wound dressings utilizing successful ostomy application Patient considered optimized for post-acute wound management

NPWT was discontinued. He was transitioned from the acute to post-acute care setting with advanced wound dressings that could ultimately be managed at home (Figures 24-25).

This patient's progress and apparent success were achieved only because of the commitment and experience of a caring group of providers that was led by their knowledge and experience of NPWT in the most challenging circumstances.

This case illustrates the multidisciplinary team approach toward a complex patient with a complex wound, with the team having worked endlessly to create new strategies using evidence-based wound therapies with negative pressure as the cornerstone of treatment.

## Discussion

NPWT is a disruptive technology that changed the standard of care for the treatment of complex wounds. More recently, the addition of instillation and dwell (NPWTi-d) has combined the benefits of NPWT with controlled delivery of topical solutions and suspensions over the wound bed, thereby reducing edema and promoting perfusion and granulation tissue formation. When used with novel dressings such as ROCF-CC, the combination facilitates the removal of thick exudates and infectious materials, such as fibrin and slough, providing a wound-cleansing option for clinicians when surgical debridement must be delayed or is not possible or appropriate [1].

NPWT has been adopted for use in inpatient and outpatient care settings for nearly all wound types due to its ability to manage wounds, promoting granulation tissues, perfusion, and reducing exudates [2].

The NPWT dressings and devices have evolved. The clinical use and management of complex wounds with NPWT has become quite common, particularly in heavily contaminated wounds. The goals of therapy while customized for each patient always include wound bed preparation as the first step to closure. Excisional debridement has been considered the gold standard for the treatment of any wound with grossly non-viable tissues [4]. In the inpatient setting, this is often accomplished in the operating room under general anesthesia. However, there are occasions when the patient is not well enough or stable enough to undergo general anesthesia, and we are looking for alternatives to assist with wound bed preparation. NPWTi-d has been shown to combine the efficacy of traditional NPWT (microstrain and macrostrain) as an effective option. Additionally, the instillation mode combined with NPWT had proven to be more effective in decreasing bacterial burden as well as achieving a faster rate of granulation tissue formation in comparison to NPWT alone [2]. In a recent advisory panel, the members reported the use of NPWTi-d with ROCF-CC (cleanse choice) as an option in patients with heavy exudates. Additionally, members reported using NPWTi-d with ROCF-CC dressings allowed for more accurate assessment demarcation of devitalized tissue. Furthermore, the continuum of care could be addressed with the continuum of NPWT by transitioning therapies from NPWTi-d with ROCF-CC dressing to ROCF-V (V.A.C VERAFLO Dressing, KCI, An Acelity Company) to NPWT with Granufoam Dressing (KCI, An Acelity Company) to NPWT incisional management with Prevena or Prevena Plus, Dressing (KCI, An Acelity Company) [1-3].

Complex wounds continue to be a challenge for all clinical specialties. A multidisciplinary team approach is often needed and/or required to achieve successful outcomes. Creating new techniques based on known benefits and mechanisms of actions can allow for continued education and sharing of information to achieve success for even the most challenging clinical scenarios.

## Conclusions

NPWT is used in all care settings. The continuum of care can be addressed with the continuum of care using NPWT. Instillation initiated in the heavily contaminated setting followed by Veraflow, followed by VAC therapy, followed by Prevena for incisional management. While other modalities and advanced wound dressings and products continue to expand and broaden our ability as clinicians to manage our patients, it is clear that NPT has become a cornerstone for treatment.

Additionally, as we continue to broaden our knowledge in the treatment of complex wounds, wound management seems to lend itself to a team approach, perhaps more so than any other clinical specialty.

Complex wounds continue to be a challenge for all clinical specialties. A multidisciplinary team approach is often needed and/or required to achieve successful outcomes. Creating new techniques based on known benefits and mechanisms of actions can allow for continued education and sharing of information to achieve success for even the most challenging clinical scenarios.

In this case, the success achieved was largely due to the willingness of all clinicians to recognize the severity of the situation as well as their limitations. Procuring help and assistance is sometimes humbling, but can be rewarding, and most often appreciated.
